# How far are we from viral hepatitis elimination service coverage targets?

**DOI:** 10.1002/jia2.25050

**Published:** 2018-04-10

**Authors:** Yvan J‐F Hutin, Marc Bulterys, Gottfried O Hirnschall

**Affiliations:** ^1^ Department of HIV and Hepatitis World Health Organization Geneva Switzerland

**Keywords:** viral hepatitis, elimination, strategy, public health, indicators, monitoring, evaluation, hepatitis B, hepatitis C, treatment access

## Abstract

**Introduction:**

In 2016, the Global Health Sector Strategy (GHSS) on viral hepatitis called for elimination of viral hepatitis as a major public health threat by 2030 (i.e. 90% reduction in incidence and 65% in mortality). In 2017, WHO's first‐ever Global Hepatitis Report presented the baseline values for each of the core indicators of the strategy. We review the challenges and opportunities that lie ahead in order to reach the 2030 service coverage targets.

**Discussion:**

Three‐dose coverage of hepatitis B vaccine in infancy reached 84% in 2015 (2030 target: 90%); however, only 39% received the timely birth dose (2030 target: 90%). Blood safety (97% of blood units screened with quality assurance, 2030 target: 100%) and injection safety (5% unsafe injections, 2030 target: 0%) had made substantial progress while harm reduction fell short (27 syringe and needle sets distributed per person who injects drugs per year, 2030 target: 300). Worldwide, 9% and 20% of the HBV‐ and HCV‐infected population respectively, were aware of their status (2030 targets: 90%). In the short term, to reach the 2020 target of diagnosing 50% of those infected, 107 million HBV infected persons and 15 million HCV infected persons should be urgently diagnosed. Overall, in 2015, less than 10% of known infected persons were on HBV treatment or had started HCV treatment (2030 targets: 80%).

**Conclusions:**

The prevention component of elimination is on track with respect to hepatitis B vaccination, blood safety, and injection safety. However, coverage of the hepatitis B vaccine timely birth dose requires a substantial increase, particularly in sub‐Saharan Africa, and harm reduction needs to be taken to scale as injecting drug use accounts for a third of mortality from HCV infection. A promising but limited start in hepatitis testing and treatment needs to be followed by immediate and sustained action so that we reach the service coverage targets required to achieve elimination by 2030. Treating persons coinfected with HIV and hepatitis viruses is particularly urgent and needs to be promoted in the context of the HIV response.

## 1 Introduction

The public health consequences of hepatitis B virus (HBV) and hepatitis C virus (HCV) infections have long been neglected 1. Till the 1980s, little had been undertaken in the field of prevention or treatment for viral hepatitis. In 1992, a World Health Assembly (WHA) resolution (WHA 45.17) called for introduction of hepatitis B vaccine in all WHO member states by 1997. This, along with financial support from the Global Alliance for Vaccine and Immunization (GAVI) and facilitated procurement of vaccines through the revolving fund of the region of the Americas led to a major increase in vaccine coverage in the 2000s 1. Prevention also took off through other initiatives on blood safety 2, injection safety 3 and harm reduction 4. However, it is only recently that more attention has been given to testing and treatment, following the availability of direct acting anti‐virals (DAAs) for HCV infection in 2014. Ultimately, in 2016, the WHA adopted the Global Health Sector Strategy (GHSS) on viral hepatitis. The GHSS calls for the elimination of viral hepatitis as a major public health threat by 2030 (defined as 90% reduction in incidence and 65% in mortality) 5. This breakthrough resolution led to subsequent resolutions by WHO regional committees and the development of regional action frameworks for hepatitis. As a result, many countries have initiated work to formulate national action plans, starting with initial assessments.

In 2014, the World Health Assembly requested the WHO Secretariat to examine the feasibility of eliminating hepatitis B and C. In 2015, the Sustainable Development Goals (SDGs) also committed to combating viral hepatitis as part of the target 3.3 6. WHO therefore commissioned a mathematical model that suggested that if the viral hepatitis response were to reach five synergistic prevention and treatment service coverage targets, hepatitis B and C could be eliminated as a major public health threat 7, 8. These five interventions are (1) hepatitis B immunization, (2) hepatitis B vaccine timely birth dose and other interventions for the prevention of mother to child transmission of HBV, (3) blood and injection safety, (4) prevention of transmission among persons who inject drugs (PWIDs) through comprehensive harm reduction and (5) testing and treatment for chronic HBV and HCV infection. Reducing the number of new infections and deaths requires a comprehensive health sector approach. It is expected that the implementation of these five priority interventions in the context of the universal health coverage framework will strengthen health systems, which is the overarching target for the Sustainable Development Goal 3 on health 9.

**Figure 1:**
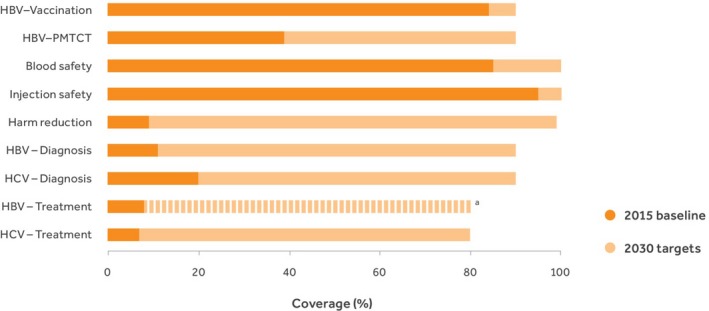
Global Health Sector Strategy on viral hepatitis: 2015 baseline towards the 2030 service coverage targets for the core interventions.

In April 2017, WHO published its first‐ever Global Hepatitis Report 1 to describe the worldwide situation in terms of viral hepatitis in 2015. The report summarizes the epidemiological situation in terms of prevalence (257 and 71 million persons living with chronic HBV and HCV infection respectively), incidence, and mortality (1.34 million deaths) in 2015. One of the objectives of this report was to estimate the baseline values of each of the core service coverage indicators of the GHSS on viral hepatitis, in relation to the proposed service coverage targets for 2030 (Figure 1). In this commentary, we review the challenges and opportunities that lie ahead for the global efforts to reach the 2030 service coverage targets that are key to achieving elimination. The core principle of the HIV/AIDS response – that no one should be denied treatment by accident of geography or income – is our overarching objective 10.

## 2 Discussion

### 2.1 Prevention

#### 2.1.1 Infant hepatitis B immunization

In 2015, the global reported coverage for the third dose of hepatitis B vaccine among infants was 84% 1, close to the 2020 (90%) and 2030 (90%) targets of the GHSS. However, there were substantial variations across WHO regions. The Western Pacific region (90%), the American region (89%) and the South East Asia regions (87%) were above the global average and close to the target of the GHSS while the European region (81%), the Eastern Mediterranean region (80%) and the African region (75%) had lower coverage. In Asia, a higher prevalence of HBV infection and higher burden from HBV‐associated cirrhosis and hepatocellular carcinoma may explain the mobilization in countries that has led to the rapid scale‐up for the control of hepatitis B through immunization 11. In the Americas, a long‐standing tradition of investment in immunization systems may explain the high reported coverage. In the European region, the situation is split. On the one hand, the European Union includes a number of countries that have not yet included hepatitis B vaccine into their routine immunization schedule. On the other hand, a number of Eastern European countries that have been going through rapid economical transition have faced difficulties in maintaining high immunization coverage. In the Eastern Mediterranean region, coverage has also been heterogeneous. In the African region, where the prevalence of chronic infection remains high in the general population, three‐dose infant hepatitis B vaccine coverage remained relatively low as a number of low‐income countries are still dependent on international development assistance to maintain immunization services and some others are facing war or civil unrest.

To achieve the 90% coverage targets of the GHSS on viral hepatitis, stronger immunization delivery systems will be needed, in the context of the Global Vaccine Action Plan 12. Most importantly, and to achieve equity, coverage exceeding 90% should be achieved not only at the global level, but also at the regional, national and sub‐national levels, and if possible, in each of the poorer districts so that no children are left behind in reducing the incidence of HBV infections in early childhood.

#### 2.1.2 Prevention of mother to child transmission of HBV infection

The current mainstay of the prevention of mother to child HBV transmission is the administration of a timely birth dose of hepatitis B vaccine (i.e. within 24 hours of birth) 13. In 2015 1, the global coverage reported was 39% (for a 2030 target of 90%). In the Western Pacific Region, coverage with the timely birth dose increased from 2% in 2000 to 83% in 2015 as a result of a commitment towards a goal for the control of HBV infection 14. In the region of the Americas, coverage also increased dramatically as a result of strong immunization services. In the African region, which is characterized by high HBV prevalence, coverage remains very low (10%), as a result of a combination of obstacles, particularly the low proportion of births that take place in the healthcare setting 15 and misconceptions among many health care staff and policymakers regarding the presumed benefit of the timely birth dose. This low birth dose coverage is problematic as those infections acquired at birth in Sub‐Saharan Africa are the ones that account for the majority of chronic liver disease later in life 16. New tools are becoming available to prevent transmission of HBV from mothers to children, including use of Tenofovir for mothers with high viral load 17. Further examination of evidence, values and preferences is needed before WHO can make a decision to recommend them for large‐scale use. In the meantime, the WHO Western Pacific Region proposes to engage in prevention of mother to child transmission of HBV infection in an incremental manner, starting with universal immunization of infants with hepatitis B vaccine (including a timely birth dose), and adding progressively and sequentially, testing and follow up and pregnant women, use of hepatitis B immune globulin and anti‐viral medicine for women with high viral load 18. Use of anti‐virals in pregnant women with high HBV viral load may be a higher priority in Asia as perinatal transmission of HBV is more common in this region 19.

#### 2.1.3 Blood and injection safety

In 2013, among the 137 countries that reported data on this indicator to the Global Database on Blood Safety, 97% were screening all blood donations using basic quality procedures, which included documented standard operating procedures and participation in an external quality assurance scheme (ahead of the 2020 target of 95%) 20. However, blood transfusion safety is still a concern, especially in low‐ and middle income countries, where the prevalence of transfusion transmissible infections is high, and quality and coverage of blood screening are inadequate 21, 22. Hence, further work is needed on the screening of blood units, in the broader context of national blood safety policies that also ensure (a) recruitment of safe, voluntary, non‐remunerated blood donors and (b) appropriate clinical use of blood.

There was substantial improvement in the safety of healthcare injections in the world between 2000 and 2010. In these ten years, the proportion of injections given with equipment re‐used in the absence of sterilization decreased from 33% to 8% 23. According to 2010 data, healthcare injections remained particularly unsafe in some countries of the Eastern Mediterranean Region, with 14% re‐use still detected and a large number of unsafe injections per capita. This persisting driver of transmission needs to be addressed through safer healthcare, introduction of reuse‐prevention devices 24 and a reduction in unnecessary healthcare injections 25, particularly in the Eastern Mediterranean and South‐East Asia regions where unsafe injection practices could lead to re‐infection after HCV cure 26. More recent and better quality data are also needed to monitor the evolution of injection safety since 2010.

#### 2.1.4 Harm reduction

Injection drug use accounts for close to a third of new HCV infections and also for about a third of the mortality from the sequelae of HCV infection 27. However, harm reduction suffers from poor documentation and very low coverage in most countries around the world. In terms of the core indicator of the GHSS for viral hepatitis (i.e. the number of syringe and needle sets distributed by person who inject drugs each year), the most recent data are from 2010 28 with an estimated 27 sets per user and per year, against 200 and 300 targets for 2020 and 2030 respectively. If viral hepatitis is to be eliminated, 29 an approach combining prevention and treatment will be needed with sufficient coverage of both 30. Integrated, high‐impact interventions recommended by WHO and other United Nations agency 31 require a policy context that prevents stigma and discrimination 32.

### 2.2 Diagnosis and treatment

Few people with viral hepatitis have been diagnosed (9% [22 million] of HBV‐infected persons and 20% [20 million] of HCV‐infected persons) 1. In the short term, to reach the 2020 target of diagnosing 50% of those infected, 107 of the 257 million HBV‐infected persons and 15 of the 71 million HCV infected persons should be urgently diagnosed. Among those diagnosed, treatment has reached only a small fraction. In 2015, 8% of those diagnosed with HBV infection were on treatment, while 7.4% of those diagnosed with HCV infection had started treatment 1. While the cumulative number of persons treated for HCV reached 5.5 million in 2015, only about half a million of these persons had received the newer, more effective and better tolerated classes of medicines called DAAs. There were more new HCV infections than patients who were started on treatment in 2015 1. Testing and treatment for viral hepatitis could build on the experience acquired with HIV in terms of health service delivery models. WHO published testing guidelines that clarify who to test and how to test 33. As the cost of treatment is rapidly decreasing in countries with access to generics, the high cost of HCV tests will become the next bottle‐neck for implementation. However, these service coverage figures have different implications for HBV and HCV.

For HBV, we lack solid estimates on the proportion of HBV infected persons that are eligible for treatment as the information on fibrosis, HBeAg status, HBV DNA values and liver function tests is often missing from epidemiological studies that report population‐based prevalence. Published studies suggest that this proportion might not exceed 10% in community‐based, rather than hospital‐based, settings 34, 35, 36. This uncertainty and the expected low proportion of persons eligible for treatment have two practical implications. First, community based testing programmes for HBV infection might be limited by a low yield in terms of persons actually eligible for treatment. Second, we cannot precisely interpret the gaps towards reaching the 2030 goal that proposes to place 80% of eligible persons on treatment. In the coming years, the availability of new treatments that could lead to a functional cure for HBV could increase the proportion of those eligible for treatment. A higher proportion of patients eligible for treatment would increase the yield of testing services and would change the perspective of the GHSS. In the meantime, patients with chronic HBV infection that meet the WHO criteria for treatment 37 can benefit from treatment with anti‐nucleos(t)ides with a high barrier to resistance, such as Tenofovir or Entecavir. The WHO guidelines 37 also make provision for the management of patients when access to HBV DNA testing is not possible. Other anti‐viral medicines that can rapidly lead to resistance, such as Lamivudine, are no longer recommended. As Tenofovir is available as a generic preparation in many countries on the international market from WHO‐pre‐qualified manufacturers for a price as low as USD 48 per year of treatment, substantial progress in terms of treatment could be achieved fast, which would lead to a short‐term impact in terms of morbidity and mortality reduction. Cost‐effectiveness studies should help to secure support from decision makers 38. In areas where hepatitis D virus (HDV) co‐infection is common (e.g. Mongolia or the Amazon in Brazil or Peru) among persons with HBV infection, testing for HDV DNA is also needed, in view of specific treatment considerations 39, 40.

For HCV, about one in five infected persons knows their status, and among those identified with infection, a large majority of patients remains untreated. Hence, the promising start of the new public health initiative to place persons on treatment needs a substantial scale‐up to place more identified persons on treatment first. The high price of DAAs has been an obstacle to scaling up treatment in many countries. However, over 100 countries can now access generic medicines for about USD 200 per curative treatment or less. At such prices, treatment is cost saving 41. Hence, appropriate and effective treatment can be provided to patients, including in most low and middle income countries, at a reasonable cost if procurement of generic medication is optimized. Increasing access to treatment requires simultaneously scaling up coverage of testing services, including to HCV RNA or HCV core antigen, which are required to make treatment decisions 33. As of 2017, the situation was evolving fast with new initiatives in Australia 1 and more persons placed on treatment in Egypt.

Among the 36.7 million persons who were living with HIV in 2015, an estimated 2.7 million had chronic HBV infection and an estimated 2.3 million had been co‐infected with HCV. Liver diseases represent a major cause of morbidity and mortality among persons living with HIV, and appropriate treatment of co‐infections with HBV and HCV has a durable impact on disease progression. If diagnosed, people should be placed on effective hepatitis treatment in the context of the HIV response.

### 2.3 Monitoring impact

WHO published a monitoring and evaluation framework to measure progress towards elimination 42. In addition to prevention indicators for which systems are usually already in place, countries will require patient databases that can be aggregated into cascades of care and surveillance for viral hepatitis, including (a) acute hepatitis, (b) chronic infections and (c) mortality from cirrhosis and hepatocellular carcinoma 43.

## 3 Conclusions

WHO's elimination strategy calls for a 65% reduction in mortality from chronic infection with hepatitis B and C viruses by 2030. A review of the 2015 baseline of the service coverage target indicators generates a contrasting picture. From a prevention perspective, global efforts are, for the most part, on track for hepatitis B vaccination, blood safety and injection safety. However, the coverage of the timely birth dose of the hepatitis B vaccine will require a major focused effort to increase coverage, particularly in Africa; and harm reduction needs to be taken to scale as injection drug use accounts for a third of HCV related mortality. With respect to testing and treatment for chronic HBV and HCV infections, there has been a promising start in a number of countries; however, access to diagnosis and treatment remains too low. Limited funding is available at the international level to support national hepatitis elimination plans. Therefore, countries will need to finance their response primarily through domestic resources. Treatment can be cost‐effective, or even cost‐saving, from a healthcare perspective 38, 41. The 2015 service coverage indicators are a clear call for immediate and sustained action so that these life‐saving services can be integrated in the universal health coverage package in order to achieve elimination by 2030. Finally, treating persons co‐infected with HIV and hepatitis viruses is particularly urgent and should be done in the context of the HIV response.

## Competing interests

The authors have no competing interests.

## Authors’ contributions

YH drafted the manuscript. MB and GH conceptualized the main messages and provided comments on the paper.

## Funding

United States Centers for Disease Control and prevention, Atlanta, GA, USA.
